# On Some Results of a Retrograde Engorgement of the Bloodvessels

**Published:** 1868-12

**Authors:** 


### ON SOME RESULTS OF A RETROGRADE ENGORGEMENT OF THE BLOODVESSELS.

My object in the present communication is to direct attention to certain results of a retrograde engorgement of the bloodvessels, consequent upon an impediment to the onward movement of the blood. Most of the facts to which I am about to refer are well-known and familiar facts; I believe, however, that they have not hitherto been collated and analyzed with the care and the accuracy which their pathological interest and their practical importance demand.

I have to speak first of certain phenomena which result from an impeded circulation through the *lungs*. I shall afterwards refer briefly to some analogous phenomena which follow an interrupted circulation through the *liver* and through the *kidney* respectively.

Let me first, then, direct attention to the well-known peculiarities of the blood-supply to the lungs. The lung, as a respiratory organ, as a great gas-secreting gland, is supplied by the pulmonary artery, the blood from which passes through the pulmonary capillaries and veins from the right to the left side of the heart. The lung, as an organ whose tissues have to be nourished, is supplied with blood from the aorta by the bronchial arteries, and this blood, having been distributed to all the proper tissues of the lung, is returned by the bronchial veins (one opening into the left superior intercostal, and the other into the vena azygos) into the superior cava. In the lung, therefore, there is the pulmonary circulation proper, and, in addition to this, there is the bronchial circulation, by means of which its own tissues are nourished, this bronchial circulation constituting a part of the general systemic circulation. "The bronchial arteries and veins carry blood for the nutrition of the lung, and are doubtless also the principal source of the mucous secretion found in the interior of the air-tubes."—(Quain's Anatomy, edited by Dr. Sharpey, 7th edition, p. 903.) It is generally believed, and it probably is a fact, that where these two different sets of vessels come into contact, where the bronchi and intercellular passages merge into air-cells, there is a communication between the two sets of vessels—between the pulmonary and the bronchial systems of vessels.

I have elsewhere sufficiently proved, that during the collapse stage of cholera, there exists a great impediment in the minute branches of the pulmonary artery. The result is, that those parts of the vascular system which lie in front of the smallest branches of the pulmonary artery are comparatively empty of blood, while the parts behind are distended. The whole of the systemic venous system is gorged with blood; hence the lividity of the face, and hence congestion of the bronchial veins and capillaries, which may impart to the lung a dark color and an appearance of congestion, while yet the lung, as a whole, contains very little blood, and is much reduced in weight. Even the *pulmonary* capillaries may become partially injected in consequence of an impeded return of blood through the bronchial veins. There is a communication between the two sets of vessels about the terminal bronchi, and a part of the blood carried by the bronchial arteries is returned by the pulmonary veins. An impeded return by the bronchial veins would therefore divert more bronchial blood into the pulmonary capillaries and veins.

Some pathologists have supposed that the impeded transit of blood through the lungs in the collapse stage of cholera is a result of its becoming too thick and viscid to pass through the capillaries. This view is proved erroneous by a number of facts, and amongst others by the anatomical fact that the mass of blood is arrested *before* it reaches the pulmonary capillaries, while the blood is obviously not too thick to enter the bronchial capillaries, which, although of a smaller size than the pulmonary capillaries, are as over-full as the pulmonary capillaries are empty of blood. In fact, there is usually a direct relation between the anæmia of the pulmonary capillaries and the engorgement of the bronchial; and the contraction of the minute pulmonary arteries, presenting an abrupt bar to the onward movement of the blood, is the probable cause of both phenomena. One result of the engorgement of the bronchial veins and capillaries during the collapse stage of cholera is a tendency to passive serous transudation through the mucous membrane into the bronchial tubes. The consequence is, that after prolonged collapse the minute bronchi and air-cells become more or less filled with serum; and when, during reaction, the blood again passes freely through the pulmonary artery, the respiratory changes are interfered with by the œdematous condition of the lung, and there often occurs a fatal apnœa.

Now let us compare the phenomena of cholera collapse with those of spasmodic asthma. The general appearance of a patient during a severe paroxysm of asthma is strikingly like that of one in the collapse of cholera. Dr. Hyde Salter, in his masterly treatise on asthma, says (p. 71): "If the bronchial spasm is protracted and intense, the heat of the body falls; the oxygenation of the blood is so imperfectly performed, from the sparing supply of air, that it is inadequate to the maintenance of the normal temperature; the extremities especially get cold, and blue, and shrunk; I have known the whole body deathly cold, and resist all efforts to warm it for four hours. But while the temperature is thus depressed, the perspiration produced by the violent respiratory efforts may be profuse, so that the sufferer is at the same time cold and sweating. It is this union of coldness and sweat, combined with the duskiness and pallor of the skin, that gives to the asthmatic so much the appearance of a dying man. The pulse during severe asthma is always small, and small in proportion to the intensity of the dyspnœa; it is so feeble sometimes that it can hardly be felt."

The resemblance between some of the most striking features of the asthmatic paroxysm and the collapse of cholera is obvious. What, then, is common to these two forms of collapse? Obviously not a drain of liquid from the blood, which was so long and so erroneously supposed to be the main cause of cholera collapse, but a partial arrest of the pulmonary circulation. In a paper which is to appear in the next volume of the "Medico-Chirurgical Transactions," I have shown that in cases of acute apnœa there is the same evidence of an arrest of blood in the minute branches of the pulmonary artery as exists in the case of cholera collapse. In both pathological conditions the pulmonary capillaries contain very little blood, while the right cavities of the heart and the pulmonary artery are distended, the left cavities of the heart being comparatively empty. In both asthma and cholera, the circulation is impeded by the muscular contraction of the small pulmonary arteries; and hence the symptoms of collapse in both diseases. In cholera, the contraction of the arteries is a result of the poisoned blood in the vessel; in asthma, it is a consequence of the partial apnœa occasioned by spasm of the bronchi. In cholera, there is a primary asphyxia and a secondary apnœa, consequent upon the blood-stasis; in asthma there is a primary apnœa, the result of bronchial spasm, and a secondary asphyxia. In both forms of disease the symptoms of collapse may speedily be removed for the time by measures which overcome the primary spasm: in asthma by the inhalation of chloroform, which relaxes the bronchial spasm; in cholera by the injection of a hot liquid into the veins, which, reaching the lungs, overcomes the arterial spasm. In asthmatic apnœa we have evidence of congestion of the bronchial system of vessels in the occasional occurrence of bronchial hæmoptysis, and in the more constant occurrence of bronchial mucous expectoration, which is copious and prolonged in proportion to the intensity and duration of the previous paroxysm.

I have dwelt at some length upon the phenomena of asthma as a type of partial acute apnœa. The causes of acute anpœa are very various; but in all essentials the phenomena are alike. Whether the apnœa be the result of hanging, drowning, suffocation, inflammatory croup, diphtheria, epileptic or tetanic spasm implicating the respiratory muscles, division of the vagi nerves as in Dr. John Reid's experiments, apoplectic coma, narcotic poisoning or injury to the upper part of the spinal cord—in each and all of these cases, in proportion to the suddenness and completeness of the apnœa, the pulmonary capillaries are anæmic, while the vessels containing black blood which lead up to those capillaries, are gorged. In all these cases, too, the retrograde passive engorgement of the bronchial veins and capillaries results in a serous and mucous, and sometimes sanguineous, exudation into the bronchial tubes.

We are now in a position to understand the condition of the lungs which is found in cases of *chronic* apnœa. In acute apnœa we have seen that the lungs are anæmic, and we have explained this by the stop-cock action of the minute pulmonary arteries consequent upon the suspension of the respiratory changes. When death occurs after a state of partial apnœa has continued for many hours, or for several days, the lungs are always found more or less gorged with blood and serum. The explanation of this is simple. The apnœal blood-stasis in the pulmonary artery throws back the blood upon the bronchial veins and capillaries, from which there is poured a serous dropsical exudation into the bronchial tubes. This fluid, sometimes blood-tinged, reaches the extremities of the bronchial tubes and the air-cells, where it compresses the network of pulmonary capillaries, and impedes the flow of blood through them. Thus the lung becomes more and more gorged with blood and serum. An analogous phenomenon may often be witnessed in anasarcous legs. The accumulation of liquid in the areolar tissue sometimes compresses the vessels so as to cause passive congestion, and not unfrequently inflammation and gangrene, of the skin and tissues beneath the skin.

A case of tricuspid regurgitation, which occurred under my care in the hospital 12 years ago, affords an instructive illustration of the effect of an impediment originating in the right side of the heart, first upon the bronchial, and secondly upon the pulmonary, circulation. I gave the particulars of this case in a clinical lecture which was published in the *Medical Times and Gazette* of February 13th, 1858. A woman, aged 52, was admitted with general dropsy, albuminuria, a systolic bellows-sound at the bottom of the sternum, distended and pulsating jugulars, and the physical signs of bronchitis. A single large dose of elaterium acting very freely upon the bowels cleared away the dropsy, the albuminuria, and the bronchial secretion. After a short time, however, *all* the symptoms returned, and she died. We found, as we had expected, great dilation of the tricuspid orifice, while the valves on the left side of the heart were normal. The lungs were much engorged. In this case it is clear that the primary cause of all the suffering was incompetence of the tricuspid valve. There was, consequently, a reflux of blood into the systemic veins; renal congestion and albuminuria, anasarca, bronchial congestion and exudation, and then a secondary obstruction of the pulmonary capillaries. It is interesting to note that before her admission she had spit some blood, the source of which was in all probability the overgorged bronchial capillaries. We are familiar enough with the occurrence of hæmoptysis as a consequence of mitral or aortic valve disease; the source of such hæmoptysis being the pulmonary capillaries, while disease on the right side of the heart throws back the strain and pressure primarily upon the bronchial vessels. If there were no bronchial vessels, if the sole blood-supply to the lungs were through the pulmonary artery, the tendency of obstruction at the right side of the heart would obviously be to render the lungs anæmic, and not to overfill them with blood.

Another interesting phenomenon, allied to those to which I have already referred, is the œdema of the sound lung, which not unfrequently occurs when one lung has been suddenly consolidated by pneumonia, or compressed by a pleuritic effusion. In such a case we sometimes hear crepitating sounds in the bronchi of the healthy lung; and we look upon this condition of things with anxiety, because we know that an increase of the œdematous effusion into the bronchi may cause fatal apnœa. Until quite recently I have always considered that the pulmonary capillaries were the source of the serous exudation in these cases. I have now no doubt that it is from the bronchial capillaries mainly that the liquid escapes. In consequence of the impervious condition of the lung on the diseased side there is a state of partial apnœa; more blood is sent to the sound lung than can readily be aërated; its progress is checked and regulated by the stop-cock action of the minute pulmonary arteries; there is more or less engorgement of the right cavities of the heart and the systemic veins; and with this there is bronchial congestion, and a consequent serous exudation into the bronchi.

Once more, it is notorious that patients who have general vesicular emphysema of the lungs are very liable to bronchial catarrh. Why is this? In an emphysematous lung there is dilation of air-cells, rupture of their walls, and obliteration of capillaries. The vascular network of the lungs is much diminished; there is consequently an impeded pulmonary circulation, fulness of the right side of the heart and systemic veins, bronchial capillary congestion, and so a liability to catarrh and bronchitis. This passive congestion of the bronchial mucous membrane predisposes to catarrh and bronchitis, just as varicose veins in the leg predispose to inflammation of the integuments. Another result of the passive congestion of the bronchial capillaries which is a consequence of emphysema, is the occasional occurrence of bronchial hæmoptysis. Of this I have seen a considerable number of instances, and not a few in which the occurrence of blood-spitting has led to an erroneous diagnosis of tubercular disease of the lung.

The *liver* resembles the lung in this respect, that, as an excretory organ, it is supplied with venous blood. Like the lung, too, it receives a separate supply of arterial blood, through the hepatic artery, for the nutrition of its own tissues. It differs from the lung in this important particular, that the blood retains its black and venous character as it emerges from the liver. There is therefore no need for a companion vein to the hepatic artery corresponding with the bronchial veins; but the blood from the hepatic artery, after supplying the liver tissues, mingles with the portal blood, and so passes on into the vena cava. In cases of cirrhosis of the liver there is an impeded circulation through the gland: the whole of the portal venous system is consequently engorged; the hemorrhoidal veins are often enlarged; there is sometimes hemorrhage from the mucous membrane of the alimentary canal, and still more frequently a serous dropsical effusion into the cavity of the peritoneum.

The *kidney* differs from both the lung and the liver in this respect, that it has but one source of blood-supply. The blood of the renal artery serves at once to nourish the gland and to supply the materials for its secretion. With respect, however, to the distribution of blood within its substance, the kidney presents some points of analogy with the lung on the one hand, and with the liver on the other. It resembles the lung in the fact that it has two sets of capillary vessels—the Malpighian capillaries and the intertubular capillaries; but it differs from the lung in the fact that the same blood passes in succession through both sets of capillaries. Then there are some points of analogy between the capillary arrangement within the kidney and that within the liver. Mr. Bowman, in his original paper "On the Structure of the Malpighian Bodies of the Kidney," pointed out that each efferent vein may be looked upon as a portal vein in miniature, the resemblance between the portal and the efferent vein consisting in the fact that each vein intervenes between two sets of capillary vessels. And thus we find that as an impediment to the circulation through the liver causes a serous exudation into the cavity of the peritoneum, from the capillary vessels in which the portal vein takes its rise, so an impeded circulation through the kidney causes a serous exudation from the capillary vessels in which these miniature portal veins—the efferent veins—originate, and this serous exudation from the Malpighian capillaries passes into the uriniferous tubes. Dr. George Robinson, Frerichs, and others have shown that a ligature on the renal vein of a rabbit causes a retrograde engorgement of the Malpighian capillaries, and a consequent escape of blood constituents into the uriniferous tubes, which, mingling with the urine, render it albuminous. In cases of Bright's disease there is an impeded circulation through the intertubular capillaries, which is, in part at least, occasioned by the pressure of the swollen tubes upon the vessels which lie between them; there is, consequently, an engorgement of the Malpighian capillaries, a serous exudation into the uriniferous tubes, and thus an albuminous condition of the urine.

We see, then, that while an impeded circulation through the lungs throws back the blood upon the bronchial veins and capillaries, and causes a serous, mucous, or sanguineous exudation into the bronchial tubes, an impeded circulation through the liver distends the portal veins and capillaries, and causes hemorrhage from the mucous membrane of the alimentary canal, or serous effusion into the cavity of the peritoneum; and, lastly, an impeded circulation through the kidney distends the Malpighian capillaries, and causes a serous exudation, and sometimes hemorrhage, into the uriniferous tubes. The results in each case are readily explained by a reference to the peculiarities of the circulation in each of these important organs—the lung, the liver, and the kidney. Without such a reference the facts are utterly unintelligible.

If there be any one who doubts whether the retrograde action of a block to the circulation can be so far-reaching as I have described it to be, he has only to be reminded of the undoubted fact, that an obstructive valvular disease, on the left side of the heart, may affect the most remote parts of the circulatory system. Thus the impediment resulting from a defective mitral valve may extend backwards through the pulmonary capillaries to the systemic veins and capillaries, causing anasarca of the feet, etc.; it may extend through the capillaries within the liver to the portal vein and its capillary origin, causing ascites; and through the intertubular renal capillaries, to the Malpighian capillaries, causing albuminuria. It is manifest that neither extreme distance from the seat of obstruction, nor the intervention of two successive sets of capillaries, will prevent that retrograde passive engorgement of vessels, which results from a block in the course of the circulating current.

In a future communication I shall endeavor to show that a careful study of the hydraulics of the circulation will lead us to the establishment of some simple, yet most useful, guiding principles for the employment of bloodletting, general and local, and for the use of hot and cold applications and counter-irritants in the treatment of inflammation.—*Lancet.*

